# Contribution of midgut bacteria to blood digestion and egg production in *aedes aegypti *(diptera: culicidae) (L.)

**DOI:** 10.1186/1756-3305-4-105

**Published:** 2011-06-14

**Authors:** Analiz de O Gaio, Desiely S Gusmão, Adão V Santos, Marília A Berbert-Molina, Paulo FP Pimenta, Francisco JA Lemos

**Affiliations:** 1 Laboratório de Biotecnologia, Universidade Estadual do Norte Fluminense-UENF, 28013-602, Campos dos Goytacazes, RJ, Brazil; 2 Instituto Federal de Educação, Ciência e Tecnologia Fluminense-IFF, 28030-130, Campos dos Goytacazes, RJ, Brazil; 3 Laboratório de Entomologia Médica, Centro de Pesquisas René Rachou, Fundação Oswaldo Cruz, 30190-002, Belo Horizonte, Brazil

## Abstract

**Background:**

The insect gut harbors a variety of microorganisms that probably exceed the number of cells in insects themselves. These microorganisms can live and multiply in the insect, contributing to digestion, nutrition, and development of their host.

Recent studies have shown that midgut bacteria appear to strengthen the mosquito's immune system and indirectly enhance protection from invading pathogens. Nevertheless, the physiological significance of these bacteria for mosquitoes has not been established to date. In this study, oral administration of antibiotics was employed in order to examine the contribution of gut bacteria to blood digestion and fecundity in *Aedes aegypti*.

**Results:**

The antibiotics carbenicillin, tetracycline, spectinomycin, gentamycin and kanamycin, were individually offered to female mosquitoes. Treatment of female mosquitoes with antibiotics affected the lysis of red blood cells (RBCs), retarded the digestion of blood proteins and reduced egg production. In addition, antibiotics did not affect the survival of mosquitoes. Mosquito fertility was restored in the second gonotrophic cycle after suspension of the antibiotic treatment, showing that the negative effects of antibiotics in blood digestion and egg production in the first gonotrophic cycle were reversible.

**Conclusions:**

The reduction of bacteria affected RBC lysis, subsequently retarded protein digestion, deprived mosquito from essential nutrients and, finally, oocyte maturation was affected, resulting in the production of fewer viable eggs. These results indicate that *Ae. aegypti *and its midgut bacteria work in synergism to digest a blood meal.

Our findings open new possibilities to investigate *Ae. aegypti*-associated bacteria as targets for mosquito control strategies.

## Background

Insects host many microorganisms that colonize and grow inside their tissues, mainly in the digestive system. These microbes are involved in various physiological functions, including food digestion, nutrition, nitrogen fixation and reproduction. Particularly, the role of midgut-associated bacteria in digestion of food has been demonstrated in several insect species [1]. These indigenous bacteria are essential sources of carbohydrases improving digestion efficiency of plant- derived polymers such as lignin, hemicellulose and cellulose, xylan and pectin [2] and may also contribute to lipid and protein digestion [3].

Bacteria associated with the gut of several mosquito species have been extensively studied from both laboratory-reared and wild populations [4-9]. Recent reports have shown that these bacteria appear to strengthen the mosquito immune system and indirectly enhance protection against malaria parasites [10,11]. However, little is known about the functional role of these microorganisms in food digestion. A previous study [7] proposed that bacteria present in *Aedes aegypti *gut ventral diverticulum could play a role in sugar metabolism processes. Their function in blood digestion has not been determined to date, although it is well known that the bacterial population increases substantially after blood feeding (ABF) [4,5,9], suggesting a potential contribution to digestive process as observed in other insects [12].

Vertebrate blood is a rich and unique source of proteins for mosquito anabolic processes, such as vitellogenesis and egg production [13]. Blood ingested is stored in the posterior midgut where proteins are digested into amino acids [14]. The lysis of RBCs, which enclose hemoglobin, the major blood protein, is the initial step of digestion [15]. Trypsin-like proteinases induced by the blood meal are responsible for the majority of blood protein digestion in *Ae. aegypti *[16]. The major trypsins are expressed only 8-12 h ABF and their different expression patterns indicate that regulation of protein digestion is highly complex [16,17]. Blood meal digestion also induces the synthesis of yolk proteins by the fat body, which is regulated by ecdysteroid hormones released by the ovary [18,19]. The yolk proteins are transported through the hemolymph and subsequently incorporated into the oocytes [20]. The highest release of ecdysone occurs about 16 h ABF while maximum vitellogenin synthesis is reached at 28 h ABF and this level is maintained until 32 h ABF [21]. After this time, the oocyte yolk continues to grow and assumes its final length 48 h ABF, stage that corresponds to the end of vitellogenesis [20,22]. The number of oocytes, or the size of a batch of eggs, determines the mosquito fecundity [23].

In this study we evaluated the contribution of gut bacteria to *Ae. aegypti *blood digestion and fecundity. Antibiotics were orally administrated to insects in order to reduce gut bacteria and provide mechanisms for assessing the functional relationship between gut microbiota and their host. To understand the role of midgut bacteria for blood digestion and egg production is of paramount importance for the development of new strategies to decrease the spread of mosquito-borne diseases.

## Results

### Effect of the reduction of midgut bacteria in the digestion of blood proteins

**Figure 1:**
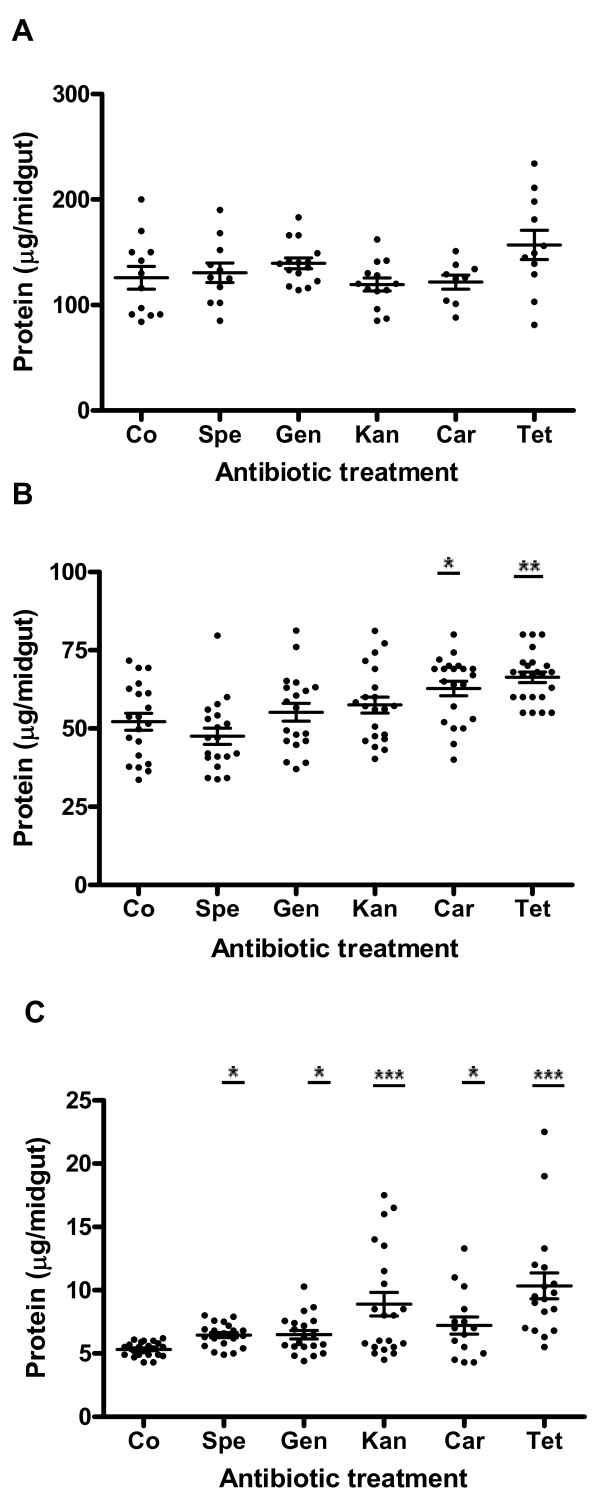
**Effects of different antibiotics on protein degradation in *Aedes aegypti *after feeding on mouse blood**. Each point represents the protein content of one mosquito midgut. A, 24 h after blood feeding; B, 36 h after blood feeding; C, 48 h after blood feeding; Co, control; Spe, spectinomycin; Gen, gentamycin; Car, carbenicillin; Kan, kanamycin; Tet, tetracycline. (*p < 0.05; **p < 0.01; ***p < 0.001). The mean ± SEM are represented by horizontal lines.

In order to evaluate if reduction of midgut bacteria could interfere with blood protein digestion, mosquitoes were treated with different antibiotics, fed on mouse and the protein content per gut was measured at 24 h, 36 h and 48 h ABF. As observed in Figure 1, there was a great variability in protein content among individuals in both control and antibiotic-treated mosquitoes. At 24 h ABF, the protein content per midgut in antibiotic-treated group ranged between 121.8 ± 6.7 μg/midgut (carbenicillin-treated mosquitoes) and 156.9 ± 13.9 μg/midgut (tetracycline-treated mosquitoes). However, these values were not statistically different when compared with the control group (125.9 ± 10.8) (Figure 1A). In contrast, at 36 h ABF, significant higher levels of blood proteins were observed in midguts of mosquitoes treated with carbenicillin (62.8 ± 2.3 μg/midgut, p < 0.05) and tetracycline (66.4 ± 1.7 μg/midgut, p < 0.01) when compared with the control group (52.2 ± 2.7 μg/midgut) (Figure 1B). Although no significant difference was noted in protein levels between gentamycin and kanamycin-treated mosquitoes and control groups, a slight tendency to higher levels was apparent. At the end of digestion (48 h ABF) no blood remained in the midguts of control mosquitoes, whereas it was common to observe red contents in midguts of antibiotic-treated ones. Mosquito midguts from all antibiotic treatments also presented levels of proteins significantly higher than the control group (5.3 ± 0.1 μg/midgut), ranging from 6.5 ± 0.2 (spectinomycin, p < 0.05) to 10.3 ± 1.0 μg/midgut (tetracycline, p < 0.001) (Figure 1C).

To determine if midgut bacteria could contribute to blood protein digestion by providing proteolytic enzymes, we investigated the effect of bacterial reduction upon the trypsin activity in the mosquito midgut. Trypsin activity assays were made in midgut homogenates from untreated and antibiotic-treated mosquitoes fed on mouse blood. Control and experimental mosquitoes presented similar mean values of trypsin activities at 12 h ABF (control, 14.8 ± 1.3 mU/midgut; carbenicillin, 16.2 ± 0.8 mU/midgut; tetracycline, 12.6 ± 1.0 mU/midgut) and 24 h ABF (control, 44.4 ± 4.1 mU/midgut; carbenicillin, 44.7 ± 3.3 mU/midgut; tetracycline, 46.7 ± 3.0 mU/midgut). Additionally, the profile of blood proteins was followed by SDS-PAGE at 12, 24 and 36 h ABF in midgut extracts from control and antibiotic-treated mosquitoes. The electrophoretic profile of blood proteins showed the same pattern in the control and treated groups, demonstrating that bacterial reduction did not affect the *in vivo *digestion of protein. Therefore, gut bacteria are not an important source for proteases capable of improving the digestion in *Ae. aegypti*.

### Impact of the reduction of midgut bacteria in *Ae. aegypti *fecundity

**Figure 2:**
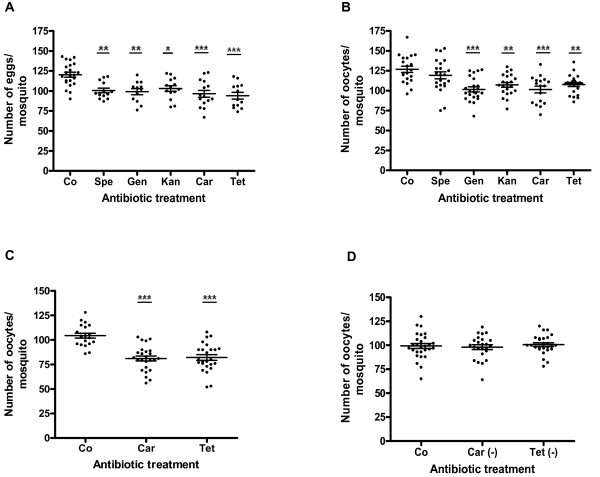
**Effects of different antibiotics in *Aedes aegypti *fecundity after feeding on mouse blood**. A, number of eggs laid by antibiotic-treated and untreated mosquitoes; B, number of oocytes in antibiotic-treated and untreated mosquitoes 48 h after blood feeding; C, number of oocytes during the first gonotrophic cycle in antibiotic-treated mosquitoes 48 h after blood feeding; D, number of oocytes during the second gonotrophic cycle in mosquitoes 48 h post-blood feeding after suspension of antibiotic treatment. Each point represents the number of eggs/oocytes in one female mosquito. Co, control; Spe, spectinomycin; Gen, gentamycin; Car, carbenicillin; Kan, kanamycin; Tet, tetracycline. (*p < 0.05; **p < 0.01; ***p < 0.001). Car (-), carbenicillin suspended on the second gonotrophic cycle; Tet (-), tetracycline suspended on the second gonotrophic cycle. The mean ± SEM are represented by horizontal lines.

Blood proteins supply the amino acids needed for vitellogenin synthesis, which is critical for egg production. Thus, we carried out assays to determine if midgut bacterial reduction could have any indirect effects on insect reproductive capacity. Either the mature oocytes or the eggs laid by each untreated and antibiotic-treated females were enumerated after feeding on mouse blood. All five antibiotics used were able to significantly reduce the number of eggs laid by each female (Figure 2A), as follows: control (120.3 ± 3.1), spectinomycin (100.5 ± 3.0, p < 0.01), gentamycin (99.1 ± 3.8, p < 0.01), kanamycin (103.0 ± 3.5, p < 0.05), carbenicillin (96.4 ± 4.3, p < 0.001) and tetracycline (94.0 ± 4.2, p < 0.001). Similar results were observed in the number of oocytes produced by each female 48 h ABF in untreated and antibiotic-treated mosquitoes (Figure 2B): control (126.8 ± 3.9), spectinomycin (119.2 ± 4.4, p > 0.05), gentamycin (101.6 ± 3.1, p < 0.001), kanamycin (107.2 ± 3.0, p < 0.01), carbenicillin (101.4 ± 4.2, p < 0.001) and tetracycline (107.7 ± 2.4, p < 0.01). As observed in Figure 2B, spectinomycin was the only antibiotic that did not reduce significantly the number of oocytes produced. Interestingly, when a cocktail containing four antibiotics (gentamycin, carbenicillin, tetracycline and spectinomycin) was administered, no additional reduction in the number of oocytes per female was observed (108.0 ± 2.9, p < 0.01).

Subsequently, we carried out assays in order to evaluate the viability of the eggs effectively laid by antibiotic- fed mosquitoes and did not observe significant differences between control and antibiotic-treated mosquitoes. More than 96% of the eggs hatched in all treatments (data not shown) showing that antibiotics did not affect the embryogenesis of the eggs. Among the tested antibiotics, carbenicillin and tetracycline were selected for additional studies because they were more effective in reducing both protein digestion process and mosquito fertility.

We also artificially fed previously antibiotic-treated mosquitoes with human blood in order to evaluate the occurrence of similar detrimental effects in fertility. As shown in Figure five A, antibiotic-treated females fed on human blood produced a smaller number of mature oocytes than the control ones. Carbenicillin and tetracycline-treated females generated 48.0 ± 2.5 (p < 0.001) and 49.3 ± 2.1 (p < 0.001) oocytes respectively, while untreated females produced 70.2 ± 1.5. These results confirmed a previous study [24], in which *Ae. aegypti *artificially fed on blood of its natural host markedly produced a lower number of eggs than the ones that fed on mouse blood.

To verify if the antibiotic effect was reversible the oocyte maturation was assessed during two consecutive gonotrophic cycles. In the second gonotrophic cycle we offered 10% sucrose solution without antibiotics to those mosquitoes that were previously treated with carbenicillin and tetracycline. As shown in Figure 2C, mosquitoes from the control group, fed on mouse blood, produced an average of 104.3 ± 2.5 oocytes/mosquito during the first gonotrophic cycle, whereas the carbenicillin and tetracycline-treated mosquitoes produced 81.00 ± 2.6 oocytes/mosquito (p < 0.001) and 82.0 ± 2.7 oocytes/mosquito (p < 0.001), representing a reduction of 22.1% and 21.1% in fecundity, respectively. During the second gonotrophic cycle, when the antibiotic treatment was ceased, the oocyte number was restored in those mosquitoes previously fed on carbenicillin (98.0 ± 2.5 oocytes/mosquito) and tetracycline (100.7 ± 1.9 oocytes/mosquito), when compared with the control group (99.3 ± 2.6 oocytes/mosquito) (Figure 2D). During the 16 days of the experiment no mortality rate was observed among experimental mosquitoes. These results demonstrated that antibiotics did not have a permanent impact upon mosquito fecundity.

### Enumeration of *Ae. aegypti *midgut bacteria after antibiotic treatment

**Figure 3:**
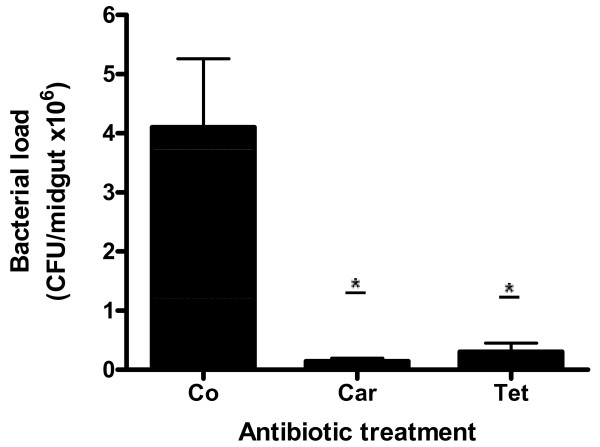
**Number of bacteria present in the midgut of *Aedes aegypti *females treated with antibiotics twenty four hours after feeding on mouse blood**. Co, control; Car, carbenicillin; Tet, tetracycline. (*p < 0.05). The mean ± SEM values were obtained from three samples containing five midguts each in two independent experiments.

We quantified *Ae. aegypti *midgut bacteria after antibiotic treatment in order to evaluate the effect of antibiotics on the growth of bacteria *in vivo*. Twenty-four hours ABF, treatments with tetracycline and carbenicillin had a dramatic effect in the midgut bacterial number, reducing 93% and 97% of viable gut bacteria, respectively. The number of colony forming units (CFU) in the control group was 4.2 × 10^6 ^± 1.2 × 10^6^, whereas in carbenicillin and tetracycline-treated mosquitoes it was 1.4 × 10^5 ^± 5.2 × 10^4 ^(p < 0.05) and 3.0 × 10^5 ^± 1.5 × 10^5 ^(p < 0.05), respectively (Figure 3).

When the antibiotic cocktail was administered to mosquitoes it reduced about 99% of the midgut bacteria (data not shown). This result was similar to the treatments using carbenicillin and tetracycline alone.

### Hemolytic activity of *Ae. aegypti *midgut bacteria

**Figure 4:**
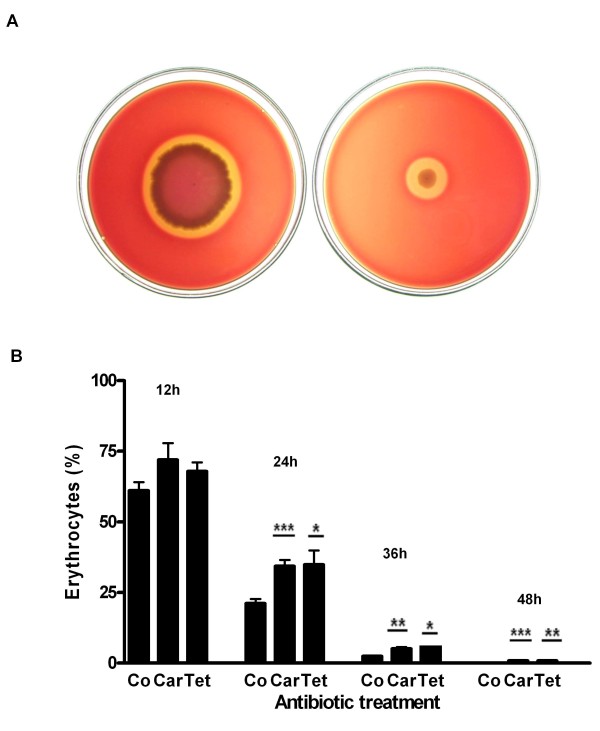
**Hemolytic activities of *Aedes aegypti *midgut bacteria during blood digestion**. A, hemolytic activities of two major bacteria isolated from *Ae. aegypti *midgut. Right, *Enterobacter sp*.; Left, *Serratia sp*. Halos correspond to the regions of erythrocytes lysis. B, percentage of erythrocytes in the midgut expressed in relation to the total ingested by recently fed mosquito (0 h). Co, control; Car, carbenicillin; Tet, tetracycline. (*p < 0.05; **p < 0.01; ***p < 0.001). The mean ± SEM values were obtained from three samples containing five midguts each in two independent experiments.

As RBCs are the main component of blood, their efficient lysis is an essential requirement for blood digestion. Therefore, we carried out *in vitro *and *in vivo *assays to assess the hemolytic activity of *Ae. aegypti *midgut bacteria. *In vitro *hemolytic activity of midgut bacterial isolates was observed in blood agar plates. The presence of a distinct translucent halo around the inoculum indicated a positive hemolytic activity (Figure 4A). The majority of bacterial isolates presented some hemolytic activity (data not shown) while *Enterobacter sp*. and all the isolates of *Serratia sp*., the major bacterial species isolated from *Ae. aegypti *midgut, showed the strongest hemolytic activity (Figure 4A).

In order to investigate bacterial contribution to the blood hemolysis *in vivo*, we followed the lysis of RBCs in mosquitoes during the digestion of mouse blood after treatment with tetracycline and carbenicillin. No significant difference was observed among the treatments 12 h ABF in spite of the higher percentage of intact RBCs remaining in carbenicillin and tetracycline-treated mosquitoes (71.9 ± 5.9% and 67.8 ± 3.3%, respectively) when compared with control mosquitoes (61.1 ± 3.0%) (Figure 4B). On the other hand, a significant difference was observed 24 h ABF, when about 35% of intact RBCs still remained in the midgut of antibiotic-treated mosquitoes (carbenicillin, 34.3 ± 2.2%, p < 0.001; tetracycline, 34.9 ± 5.0%, p < 0.05) and only 21% of intact RBCs were observed in the midgut of control group (21.1 ± 1.7%). A similar pattern of difference was observed 36 h ABF. A small number of intact RBCs was still observed in the lumen of antibiotic-treated females, while RBCs were not seen in non-treated females 48 h ABF (Figure 4B).

**Figure 5:**
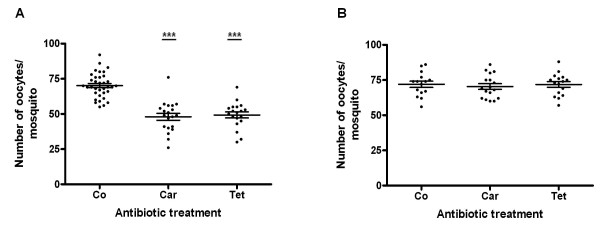
**Effect of carbenicillin and tetracycline on *Aedes aegypti *fecundity 48 h after feeding on human blood**. A, mosquitoes artificially fed on human blood. B, mosquitoes artificially fed on human blood containing 50% of lysed erythrocytes. Each point represents the number of oocytes in one female mosquito. Co, Control; Car, carbenicillin; Tet, tetracycline. The mean ± SEM are represented by horizontal lines.

The importance of midgut microbiota to blood hemolysis was finally investigated by feeding untreated and antibiotic-treated mosquitoes with human blood containing 50% of lysed RBCs. As observed in Figure 5B, the reduction of bacteria in the midgut did not affect the production of oocytes in females fed on the partially hemolyzed blood. The mean values obtained in this assay were the followings: 72.0 ± 2.2 (control), 71.8 ± 2.0 (carbenicillin), and 70.4 ± 2.0 (tetracycline). Therefore, based on the results herein obtained, it is possible to affirm that *Ae. aegytpi *gut bacteria are indeed involved in the lysis of RBCs.

## Discussion

Insects that strictly depend upon blood throughout their lives are generally in close association with symbiotic microorganisms [25]. These symbionts provide nutritional components to their host, such as B vitamins, which are scarce in vertebrate blood. The elimination of obligate symbionts results in retarded growth and a decrease in fecundity [26,27]. In contrast, mosquitoes lack a typical obligate symbiont probably because they are polyphagous during their life cycle, being detritivores in the larval stage and requiring blood and a sugar source during the adult stage. Recently, bacteria of the genus *Asaia *have been described as symbionts in *Anopheles stephensi *[6] and *Ae. aegypti *[28]. These bacteria seem to be important to these insects because they colonize their gut and other tissues and are vertically transmitted [6,28]. Nevertheless, a physiological role has not been attributed to *Asaia *or other bacterial species identified in mosquitoes to date.

In a previous work [9] we observed that intestinal bacteria presented an unequal distribution along the insect midgut during the first 24 h ABF, prevailing in regions rich in proteolytic activity. These findings, associated with the exponential growth of bacteria during the first 48 h ABF, suggest a significant importance of intestinal bacteria for blood digestion in *Ae. Aegypti*. Accordingly, in this study we carried out several experiments to test the hypothesis previously proposed in [9].

We orally administrated five different antibiotics to reduce mosquito gut microbiota and assessed their functional role in *Ae. aegypti *blood digestion. As blood is rich in proteins, an important putative role of midgut bacteria would be to help mosquitoes to digest these proteins by participating in the synthesis of digestive proteases. During blood digestion *Ae. aegypti *produces mostly trypsin-like enzymes which are expressed in two well defined phases [29]. The early phase, from 0 to 12 h ABF and the late phase, from 12 to 48 h ABF, are characterized by a small and a large increase in trypsin activity, respectively [16].

We made enzymatic assays with BApNA, which is a specific substrate for trypsin, using midgut homogenates from untreated and antibiotic-treated mosquitoes. The bacterial reduction did not affect the activity of trypsin-like enzymes in the midguts of *Ae. aegypti *during the early and late phases of digestion. Therefore, it was possible to conclude that bacteria did not synthesize either trypsin-like enzymes or other types of proteases since midgut homogenates from control and antibiotic-treated samples presented the same SDS-PAGE protein profiles. However, even maintaining the same level of proteolytic activity after bacterial reduction, the digestion of blood proteins was still delayed in mosquitoes treated with antibiotics. A significant increase in protein levels was observed at 36 h and 48 h ABF in the midguts of antibiotic-treated mosquitoes. Considering that hemoglobin is the major blood protein, its release from RBCs is an important step for blood digestion. Thus, another putative functional role of midgut bacteria would be helping in the lysis of RBCs. To verify this hypothesis, we initially tested if *Ae. aegypti *indigenous bacteria were capable of lysing RBCs through *in vitro *assays. Our results showed that hemolytic activity was a common characteristic among isolates of midgut bacteria. It is important to note that *Enterobacter sp*. and *Serratia sp *isolates, the major bacteria associated with *Ae. aegypti *to date [9], presented strong hemolytic activity. Both bacteria are able to produce molecules with hemolytic activity [30-33].

In order to verify if midgut bacteria could play a role in the hemolytic process *in vivo*, we followed the lysis of RBCs in antibiotic-treated and untreated mosquitoes during the digestion of mouse blood. It was possible to observe that midgut bacteria were already contributing to blood hemolysis at 12 h ABF, although the lysis of RBCs was not statistically significant. The antibiotic treatment significantly reduced the intensity of RBC lysis at 24 h, 36 h and 48 h ABF. During this phase of digestion, under normal conditions, bacterial populations increase exponentially, reach their maximum number and occupy the entire midgut lumen [9]. Accordingly, it was observed that RBC lysis in *R. prolixus *gut followed a similar profile of bacterial growth during blood digestion [33]. We carried out an additional experiment to clarify the role of gut bacteria in the lysis of RBCs. Control and antibiotic-treated mosquitoes were fed on human blood containing half of the RBCs already lysed. We observed that experimental mosquitoes produced a similar number of oocytes as the untreated ones, showing that midgut bacteria indeed contributed to the effective lysis of RBCs. We were then able to conclude that when female fed on blood containing a sufficient amount of hemoglobin already available, the digestion of blood occurs normally, independently of the presence of bacteria.

In contrast to the results obtained in the hemolysis assays, we did not observe differences in protein levels between antibiotic-treated and control group 24 ABF. Probably, the protein determination method did not distinguish the partially hydrolyzed hemoglobins and peptides from the intact hemoglobins inside the RBCs. The intact hemoglobins were released at the moment the reagents were added to the samples. The significant differences observed at 36 and 48 h ABF may be explained by the fact that most peptides were probably hydrolyzed into amino acids and were already transported to the midgut cells and hemolymph.

Since *Ae. aegypti *is an anautogenous mosquito, we were already expecting that the digestion of blood would be strictly connected with vitellogenesis. We found a reduction of 14-22% in egg production and 6-20% in oocyte maturation when females were treated with different antibiotics before the mouse blood feeding. A reduction of 30% in oocyte number was obtained when mosquitoes were fed on human blood, indicating that midgut bacteria are even more efficient when *Ae. aegypti *feeds on its natural host. A similar reduction in fecundity (31%) was obtained in a study about RNAi silencing of late phase trypsins expression in *Ae. aegypti *[16]. In our study in the late phase proteolytic enzymes were available for digestion; however, the amount of available hemoglobins was reduced. At this stage, enzymes and proteins must be promptly accessible in order to the digestion process be effectively completed, allowing the female to produce a normal batch of eggs.

The conversion of blood proteins into amino acids is essential for the insect reproductive cycle [19,34]. Amino acids are believed to act as signals to activate vitellogenesis and are critical for the development of eggs [19]. However, the negative effects of antibiotic treatment were not permanent since the reproductive capacity of mosquitoes was re-established during the second gonotrophic cycle. Antibiotics must have been either degraded during blood meal or excreted in the faeces. Probably, the remaining bacteria multiplied quickly after the optimal conditions were re-established. Some studies have reported that adult mosquitoes already emerge with some bacteria established in them, mainly in the gut, and also are capable of dramatically increase their number upon the acquisition of a blood meal [9,35].

The functions of *Ae. aegypti *gut microbiota still remain poorly understood. However, based on the results reported here and in previous works [7,9], we are convinced that *Ae. aegypti *and its indigenous gut bacteria have established a synergistic relationship in order to accomplish the blood digestion more efficiently.

## Conclusions

We propose that the reduction of midgut bacteria affects blood digestion, reducing the availability of protein mass to mosquitoes due to a slower lysis of RBCs. This conclusion is consistent with the experiments done by [36] where they showed that *Ae. aegypti *fecundity is dependent on blood quality, which is proportional to RBCs' levels in blood meal. Our study goes further by showing that even those bacteria that are not obligate symbionts have important implications in blood digestion and, consequently, in mosquito fertility.

The repeated cycles of blood feeding required by anautogenous mosquitoes make them efficient vehicles of spreading pathogens from host to host [19]. Therefore, a comprehensive study of the microorganisms associated with mosquitoes would provide an invaluable database for the development of new control strategies. The reduction of fecundity, through inhibition of blood meal digestion, could be an alternative control strategy for *Ae. aegypti*, decreasing frequencies of pathogen transmission.

## Methods

### Mosquito rearing

Insects were obtained from colonies of *Ae. aegypti *(Rockfeller strain) and maintained as described in [9]. Production of eggs was induced after providing mouse blood meal for insects. Females laid eggs on moisture filter paper 48-72 h ABF. After 48 h desiccated eggs were transferred to plastic containers filled with distilled water for larvae eclosion.

### Mosquito feeding

Mice (CF1 strain) were used to feed female mosquitoes. The artificial feeding was done as follows: fresh human blood was collected and submitted to three cycles of freezing and thawing. Then the hemolyzed blood was added to intact human blood in order to obtain a final concentration of 50% lysed RBCs. Either the intact or the partially hemolyzed human blood was offered to female mosquitoes through a membrane-feeding apparatus consisting of an artificial membrane stretched across the base of a water-jacketed glass cylinder at 37°C.

### Antibiotic treatment

Initially, cages were wiped with 70% ethanol before introducing the pupae. During the whole experiments emerged adult mosquitoes were daily fed on fresh 10% sterile sucrose solution mixed with one of the following antibiotics: gentamycin, carbenicillin, spectinomycin, kanamycin and tetracycline (200 μg/mL, Sigma Chemical Co., St. Louis, Mo). Females from control group were fed on 10% sterile sucrose solution without antibiotic. Mosquitoes were allowed to feed on sucrose solution for three consecutive days and then received either mouse blood or human blood.

### Protein determination

Midguts from untreated and antibiotic-treated mosquitoes were dissected out and homogenized in 100 μL of distilled water at 24, 36 and 48 h ABF. The homogenates were kept at -80°C until used. Protein quantification was determined according to the method described in [37], using bovine serum albumin as a standard. One midgut was used in at least ten independent replicates.

### Trypsin activity and polyacrylamide gel electrophoresis

Serine-proteinase activity was determined as described in [38] using N-α-benzoyl-l-Arg-p-nitroanilide (l-BApNA) as substrate at a final concentration of 0.87 mM in 50 mM Tris-HCl buffer (pH 8.0). SDS-Polyacrylamide gel electrophoresis (SDS-PAGE) was performed according to the method described in [39]. Samples were loaded into a gel composed of stacking gel (5% w/v) and resolving gel (12% w/v) and were run in a Mini-Protean II Cell electrophoresis unit (Bio-Rad, Hercules, CA, USA) at 100 V for 2 h. Assays were performed using midguts from untreated, carbenicillin and tetracycline-treated females 12 and 24 h ABF (trypsin activity) or 12, 24 and 36 h ABF (SDS-PAGE). The assays were done in triplicates and repeated three times.

### Bacterial enumeration

Twenty-four hours ABF, untreated, tetracycline and carbenicillin-treated mosquitoes were surface sterilized by immersing them for one minute in 70% ethanol and rinsed three times in sterile phosphate buffer saline (PBS). Mosquitoes were dissected under a stereoscopic microscope using a double cavity glass slide containing sterile PBS. Five midguts were rinsed in sterile PBS and transferred into a 1.5 mL tube containing 100 μL of PBS. The tube content was mixed thoroughly with a pestle and serially diluted (10^-1 ^through 10^-7^) and an aliquot of 100 μL of each tube was transferred to Petri dishes containing BHI agar. Plates were incubated at 28°C for 24-48 h. Then, bacterial colonies were counted and reported as colony forming units (CFU/mL). This assay was done in triplicates in two independent experiments.

### Hemolytic activity of bacteria in human blood agar plate

Hemolytic activity was verified through inoculation of bacterial isolates from the main genera identified in *Ae. aegypti *[7,9] in brain heart infusion (BHI) agar with 4% human blood. Microorganisms were inoculated individually in the center of the plate with a sterile wooden stick. After 48 h at 28°C, plates were observed to assess the formation of lysis zones (halos) around the inoculated bacteria.

### Red blood cell enumeration

The enumeration of RBCs was done at 12, 24, 36 and 48 h ABF. Five midguts from untreated, tetracycline and carbenicillin-treated mosquitoes were transferred to 1.5 mL plastic tube containing 100 μL of 0.3 M sucrose and gently homogenized by pipetting up and down 10-times using a Gilson pipette (P-200). Subsequently, 10 μL of a diluted aliquot was transferred to a haemocytometer. The number of RBCs obtained in each treatment and control group was compared to that obtained from females dissected immediately ABF. Cell counts were made in triplicates in two independent experiments.

### Fecundity and viability assessment

Untreated or antibiotic-treated female mosquitoes were fed either on mouse blood or human blood followed by enumeration of oocytes or eggs. Forty-eight hours ABF a sample of females was dissected under a stereoscopic microscope for oocytes enumeration and another sample was individually transferred to 50 mL plastic tubes containing a moisturized filter paper strip for enumeration of eggs. Females were allowed to oviposit for 48 h. After oviposition, the filter paper strips were removed and dried at room temperature (25°C) for three days. The number of eggs laid per female on the filter paper was then counted under a stereoscopic microscope. Egg viability was determined by submerging pieces of filter paper containing about 200 desiccated eggs from each of the treatments in 200 mL of deoxygenated-distilled water and the number of total and hatched eggs was recorded after 24 h under a stereoscopic microscope.

### Fecundity of mosquitoes during the first and second gonotrophic cycles

Effect of antibiotics on oocyte maturation was assessed during two gonotrophic cycles. Initially, female mosquitoes were fed on fresh sterile 10% sucrose solution containing 200 μg/mL of either tetracycline or carbenicillin for three days. Subsequently, mosquitoes were fed on blood mouse and one group was allowed to lay eggs, whereas the other was dissected 48 h ABF in order to count the number oocytes (first gonotrophic cycle). Then, for the second gonotrophic cycle assessment, the remaining insects were maintained on fresh sterile 10% sucrose solution without antibiotics and were blood-fed again four days later. Forty eight hours ABF, the number of oocytes was counted. During the whole assay control group was maintained under the same conditions as the treatments, except that it was not treated with antibiotics.

### Statistical analysis

Protein concentrations, eggs and oocytes enumeration were statistically analyzed by Kruskal-Wallis test and the unpaired Student's t-test was used to compare the means of RBC percentages and trypsin activities using GraphPad Prism statistical software package (Prism 5.01; GraphPad Software, Inc., San Diego, CA). Asterisks indicate significant differences (*p < 0.05; **p < 0.01; ***p < 0.001).

### Ethical approval

Approval for feeding mosquitoes on mouse was obtained from the University of North Fluminense Ethical Committee.

## Competing interests

The authors declare that they have no competing interests.

## Authors' contributions

FJAL, AOG, DSG and MABM designed the study. AOG and DSG performed the experimental work. FJAL, AOG, PFPP, DSG, AVS and MABM analyzed the data. FJAL and AVS prepared the manuscript with the critical input of AOG, PFPP, MABM and DSG. All authors read and approved the final manuscript.
